# Effect of Nanoparticles on Protein Folding and Fibrillogenesis

**DOI:** 10.3390/ijms10020646

**Published:** 2009-02-20

**Authors:** Li Fei, Sarah Perrett

**Affiliations:** 1 National Laboratory of Biomacromoleules, Institute of Biophysics, Chinese Academy of Sciences, 15 Datun Road, Chaoyang District, Beijing 100101, China; 2 Graduate University of the Chinese Academy of Sciences, 19 Yuquan Road, Shijingshan District, Beijing 100049, China; E-Mail: lfeish@hotmail.com

**Keywords:** Nanoparticles, protein folding, fibrillogenesis, amyloid

## Abstract

The large surface area and small size of nanoparticles provide properties and applications that are distinct from those of bulk materials. The ability of nanoparticles to influence protein folding and aggregation is interesting, not only because of the potential beneficial applications, but also the potential risks to human health and the environment. This makes it essential that we understand the effect of nanoparticles on fundamental biological process, like protein folding. Here, we review studies that have examined the effect of nanoparticles on protein folding and aggregation, providing insight both into the mechanisms of these processes and how they may be controlled.

## 1. Introduction

Nanotechnology has experienced rapid growth in recent years with its broad application in drug delivery, imaging and diagnosis [1–6]. Nanoparticles are materials at sub-micrometer scales, usually 1–100 nm, so they possess a large surface to volume ratio. Generally, nanoparticles are composed of two parts: the core material, and a surface modifier that may be employed to change the physicochemical properties of this core material [6]. The core materials may be biological materials like peptides, phospholipids, lipids, lactic acid, dextran or chitosan, or may be formed of a chemical polymer, carbon, silica, or metals [6–8]. Nanoparticles have significant adsorption capacities due to their relatively large surface area, therefore they are able to bind or carry other molecules such as chemical compounds, drugs, probes and proteins attached to the surface by covalent bonds or by adsorption. Hence, the physicochemical properties of nanoparticles, such as charge and hydrophobicity, can be altered by attaching specific chemical compounds, peptides or proteins to the surface [6,7,9,10]. The functionality of nanoparticles is thus enhanced or changed. The efficacy of nanoparticles for any application depends on the physicochemical characteristics of both their core material and surface modifiers. The core composition of the nanoparticles, together with their surface properties, also determines their biocompatibility and their ability to be biodegraded.

Apart from the impact on function, modifiying the surface properties of nanoparticles is also used to reduce their toxicity. With the rapid growth of nanotechnology, there are growing concerns about the toxicity of nanoparticles especially in situations where these nanoparticles serve as drug carriers [2,11]. Direct interaction of nanoparticles with human body fluids, which include many different kinds of proteins, could potentially cause unwanted biological responses. Detailed information about these interactions is still lacking. Proteins are important biological molecules that are fundamental to the proper functioning of cells and organisms, therefore the impact of nanoparticles on living organisms at the protein level is a critical issue that is attracting increasing attention. Here, we review emerging studies on how nanoparticles impact protein folding and aggregation, thus contributing to the understanding of mechanisms of folding and aggregation, while shedding light on both beneficial applications and potential harmful effects of nanoparticles.

## 2. Conformational changes occur when proteins adsorb onto nanoparticles

While binding of proteins to planar surfaces often induces significant changes in secondary structure, the high curvature of nanoparticles can help proteins to retain their original structure. However, study of a variety of nanoparticle surfaces and proteins indicates that the perturbation of protein structure still happens to varying extents. The unfolding kinetics of lysozyme or β-lactoglobulin when adsorbed onto silica nanoparticles shows that upon adsorption, the proteins show a rapid conformational change at both secondary and tertiary structure levels [12,13]. Many studies have found that loss of α-helical content occurs when proteins are adsorbed onto nanoparticles, with or without an increase in β-sheet [12–14].

Bovine serum albumin (BSA) readily undergoes conformational changes, for example, at different values of pH. Hence, BSA provides a good model for investigating the effect of nanoparticles on protein conformational change. It was found that in the presence of gold nanoparticles, BSA shows a decrease in α-helical structure, as detected by circular dichroism, and a significant increase in sheet and turn structures, as detected by Fourier transform infrared (FTIR) spectroscopy [12]. Although the protein may retain most of its native structure after adsorption on the nanoparticle surface, in some cases the thermodynamic stability of the protein is decreased, making the protein more sensitive to chemical denaturants such as urea [15]. Hence, concerns have been raised about the application of protein-nanoparticle conjugates, as changes in protein structure or stability may lead to loss of biological function. More importantly, the application of nanoparticles in drug delivery is challenged by the potential destruction of protein function when nanoparticles enter the human body. Therefore, it is essential to understand in detail the effects of protein-nanoparticle adsorption, including identifying which properties of nanoparticles determine their tendency to perturb protein conformation. In general, structural changes occur due to the intrinsic properties of the protein combined with features of the nanoparticles, such as surface chemistry and surface curvature. In addition, recent studies have characterized how the effect of protein concentration at the nanoparticle surface influences effects on protein structure and function [12,13].

## 3. Size of nanoparticles influences both structure and function of adsorbed proteins

The size of nanoparticles determines the surface curvature: this means that nanoparticles of large size have low surface curvature, while those of small size possess high surface curvature, and curvature is lowest for a planar surface. A systematic study of the effect of nanoparticle size on the structure and function of adsorbed proteins has been performed, in which lysozyme adsorbed onto silica nanoparticles was used as a model system [14]. This study conclusively showed that smaller nanoparticles more strongly favor native-like protein structure, resulting in higher intrinsic enzyme activity. Upon adsorption onto nanoparticles, lysozyme was found to undergo a reduction in both α-helical content and enzymatic activity, with greater loss on larger nanoparticles. Similar results have since been observed for RNase A adsorbed onto silica nanoparticles: a greater decrease in the thermodynamic stability of RNase A was observed when the protein was adsorbed onto larger nanoparticles [15].

**Figure 1.**
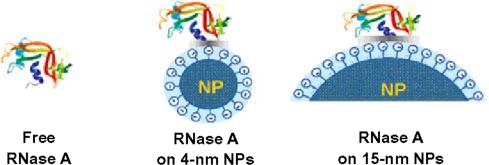
Schematic for the interaction of RNase A with silica nanoparticles of different diameters (reproduced from [15] with permission © 2007 American Chemical Society).

The mechanism behind the impact of different sizes of nanoparticles on protein structure can be explained by a simple model (Figure 1). Larger nanoparticles provide larger surface area of contact for adsorbed proteins which results in stronger interactions between proteins and nanoparticles. The greater degree of interaction leads to greater perturbation of protein structure.

## 4. Surface concentration affects the extent of protein conformational changes

Different surface concentrations can be obtained by keeping the protein concentration constant and varying the amount of nanoparticles added to an aqueous solution of protein. Higher surface concentration means that more proteins are adsorbed on the nanoparticle surface, and a crowded surface environment facilitates protein-protein interactions. In contrast, at lower surface concentration, interactions between the protein and the nanoparticle surface will predominate. Recently, studies by Wu and colleagues showed that both β-lactoglobulin and lysozyme unfolded to a greater extent at lower surface concentration on silica nanoparticles than when the surface concentration of protein was higher [13,16]. This can be explained by the intrinsic stability of the protein in a protective crowded surface environment. In contrast, at lower surface concentration, interactions are readily formed between the protein and the hydrophobic surface of the silica nanoparticles, which reduces the net energy barrier to unfolding. From this interesting finding, we can infer that immobilizing enzymes onto silica nanoparticles at high packing density will lead to less loss of enzymatic activity than if lower surface densities are used.

## 5. Chaperone activity of nanogel nanoparticles

Although it is widely accepted that after adsorption onto nanoparticles, protein structure will be perturbed to varying extents, it is also interesting to address whether some nanoparticles may act as chaperones, to improve the efficiency of protein folding. When a protein is in its native state, its hydrophobic core is buried, and the protein surface is generally rich in hydrophilic residues, which interact favorably with the aqueous environment. Upon unfolding, exposure of hydrophobic surfaces readily leads to protein aggregation. Chaperones, like GroEL and GroES, selectively bind to unfolded proteins through hydrophobic or electrostatic interactions, stabilize them from aggregation, and help them fold to the native conformation [17,18]. Chaperones are frequently applied in biotechnology for obtaining biologically active recombinant proteins [19].

**Figure 2.**
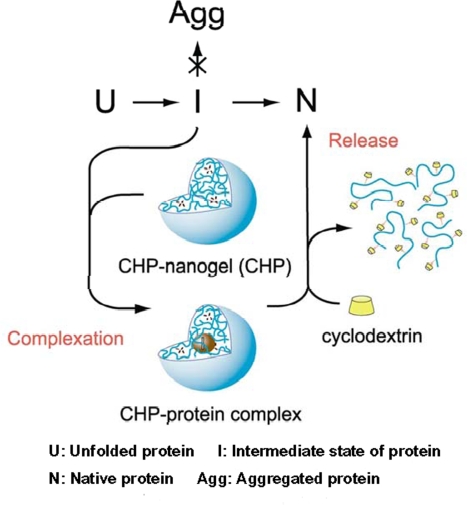
Schematic representation of artificial molecular chaperones (reproduced from [21] with permission © 2003 Elsevier).

Currently, two types of nanoparticles have been found to help protein refolding. A nanogel, formed by the self-aggregation of pullulan bearing a cholesterol group (CHP), behaves as a molecular chaperone to completely prevent irreversible aggregation upon heating of carbonic anhydrase B (CAB) by forming a complex with the enzyme. Upon the addition of β-cyclodextrin to the complex, the trapped CAB is released and its refolding occurs upon dissociation of the CHP-nanogel [20]. A schematic representation of how this artificial molecular chaperone helps refolding of CAB is shown in Figure 2. In this case, CHP acts as a host to trap unfolded proteins or intermediates during refolding to prevent aggregation, in a similar manner to GroEL, while β-cyclodextrin behaves like a co-chaperone or ATP to trigger the release of proteins in the refolded form. A later study further demonstrated the chaperone-like activity of this hydrogel nanoparticle. The nanogel prevents protein aggregation for both CAB and Citrate synthase when refolded after GdmCl denaturation; upon the addition of β-cyclodextrin, more than 70% of enzyme activity could be recovered. Interestingly, the nanogels selectively bind the CAB refolding intermediates rather than its native form [21]. Moreover, this CHP nanogel can also induce a conformational alteration of Aβ from a random coil to α-helix or β-sheet-rich structure to inhibit the fibril formation of Aβ molecules [22].

**Figure 3.**
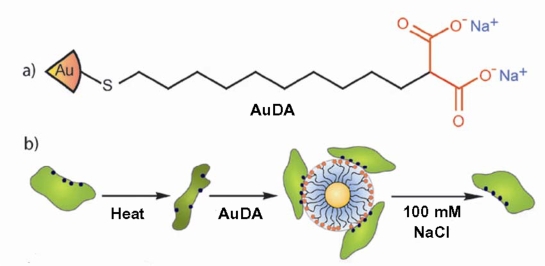
**(a)** Schematic representation of the structure of the AuDA and **(b)** thermal denaturation followed by nanoparticle mediated refolding of proteins (reproduced from [23] with permission © 2008 The Royal Society of Chemistry).

Another nanoparticle shown to have chaperone-like properties are 2 nm core gold nanoparticles functionalized with 2-(10-mercaptodecyl)malonic acid (AuDA), which bear a high negative charge density [23]. Cationic proteins, such as α-chymotrypsin, lysozyme and papain can be rescued from their thermally unfolded states by addition of AuDA. AuDA can form a complex with cationic proteins due to electrostatic interactions, thus preventing aggregation and assisting refolding (Figure 3). After the addition of 100 mM NaCl, proteins are released from AuDA, and the restoration of enzymatic activity indicates successful protein refolding.

## 6. Nanocrystals are used as a FRET probe to detect protein-folding intermediates

Understanding how proteins fold is traditionally a difficult problem in biology. A number of advanced techniques are emerging to decipher the rules of protein folding. Although progress has been made during past decades, much remains to be learned. The rapid development of nanotechnology has influenced research on protein folding.

It has been reported recently that semiconductor nanocrystals of CdS can be covalently attached to human serum albumin (HSA) as a nanoprobe in a Forster resonance energy transfer (FRET) assay to study the thermally unfolded states of HSA [24]. The function of nanocrystal CdS in this case is just like organic fluorophores, which are commonly used in FRET assays as energy donors or acceptors. Compared with conventional organic dyes, nanocrystals have tunable emission, exceptional photostablity, higher photoluminescence lifetime and higher quantum yield. These features make nanocrystals a useful tool for further study of protein folding by FRET.

## 7. Effect of nanoparticles on fibrillogenesis

As mentioned above, the interaction of nanoparticles with proteins can cause perturbation of both protein structure and function. Therefore, it is not surprising that interaction of nanoparticles and amyloid proteins or peptides might inhibit or facilitate amyloid formation. The effect of nanoparticles on fibrillogenesis is drawing growing attention. Protein or peptide fibrillogenesis is associated with amyloid diseases such as Alzeimer’s and Parkinson’s [25]. Currently, there are no effective drugs to cure these diseases and treatment options are extremely limited [26]. Engineered nanoparticles could be potential drugs for controlling or curing amyloid-related diseases if designed to be biocompatible and biodegradable, and if safety issues can be addressed.

Nanoparticles are so small that they can access almost every part of the human body, even passing through the blood brain barrier [27]. Thus, if nanoparticles can inhibit the process of fibrillogenesis or disaggregate amyloid fibrils [28], they have great potential to be used as drugs to control neurodegenerative amyloid diseases like Alzeimer’s. At the same time, detailed information about the interactions between nanoparticles and amyloid proteins could help to elucidate the mechanism of fibril formation at the molecular level, an area which still remains elusive. On the other hand, the toxicity of nanoparticles has become a topical issue as nanoparticles are being applied increasingly in medical applications [3–6]. If nanoparticles facilitate the process of protein fibrillogenesis, this presents a serious risk to human health, and great care would have to be taken to ensure their safe use.

Aβ peptides are widely used as model proteins to investigate the effect of nanoparticles on fibrillogenesis and the potential application of nanoparticles in treating neurodegenerative diseases. Most studies indicate that it is the composition of nanoparticles and their surface characteristics, rather than surface curvature or surface concentration, which determine their impact on fibrillogenesis. In the presence of PEGylated phospholipid nanomicelles, Aβ-42 exists in a predominantly α-helical conformation thereby reducing its aggregation potential [29]. Fullerene was found to inhibit significantly the amyloid formation of Aβ-40 by specifically binding to its central hydrophobic motif, KLVFF [30]. Fluorinated nanoparticles were shown to induce helical structure in Aβ40 leading to no peptide aggregation, whereas hydrogen nanoparticles accelerate the conversion of Aβ40 from random coil to β-sheet resulting in fibrillogenesis [31].

While some scientists are working hard to design nanoparticles which could be used as drugs to inhibit fibril formation or disrupt fibrillar aggregates [28], *in vitro* experiments indicate that some nanoparticles actually accelerate fibril assembly kinetics, leading to concerns about the risks of increasing environmental exposure to nanoparticles [32,33]. The nucleation process, which is the rate-determining step in fibrillogenesis, is highly dependent on peptide concentration. Monitoring by surface plasmon resonance (SPR) has shown that β2-microglobulin forms multiple layers on *N*-isopropylacrylamide/*N*-*tert*-butylacrylamide (NIPAM/BAM) nanoparticles, which means a high local protein concentration favorable for amyloid fibril formation is produced [33,34]. TiO_2_ nanoparticles have been found to promote β-amyloid fibril formation *in vitro* by the same mechanism: these nanoparticles possess strong adsorption capacity, leading to a high local concentration of Aβ peptide on the nanoparticle surface, which results in a shortened lag-time for Aβ assembly into fibrils. Therefore, it seems that a high local protein concentration explains the mechanism by which nanoparticles can facilitate fibril formation. However, a high local concentration of protein on the nanoparticle surface can also be used to explain why some nanoparticles inhibit fibril formation: binding between nanoparticles and amyloid proteins could block active sites for fibril formation and also lead to low protein concentration in solution, thus causing inhibition of fibril formation [35]. Therefore, more information is needed to evaluate the possible impact of nanoparticles on fibrillogenesis.

From the data obtained from *in vitro* experiments, it is hard to predict the behavior of nanoparticles in the human body. For example, under *in vitro* conditions, TiO_2_ nanoparticles have been found to promote Aβ fibrillogenesis. However, in the human body, the concentration of TiO_2_ would be much lower, probably under 0.0001 mg/mL, at which concentration no effect was detected even *in vitro* [32]. Further, a large number of other proteins could compete with Aβ peptide for interaction with nanoparticles, which would reduce the accessibility of the nanoparticles to Aβ peptide and reduce the likelihood of producing a high surface concentration of the amyloidogenic peptide.

In studying the impact of nanoparticles on fibrillogenesis, no general trend of responses has been observed. Current practices for evaluating the impact of nanoparticles still rely on a case-by-case approach, which is laborious and time-consuming due to the large variety of nanoparticles [34].

## 8. Conclusions

*In vitro* research on how nanoparticles affect protein folding and aggregation, as summarized here, is shedding light on how nanoparticles may interact with proteins when they enter the human body. However, whether the presence of nanoparticles will disrupt normal protein function and cause adverse side-effects, and whether nanoparticles will function as intended *in vivo,* remains to be seen. Detailed characterization of plasma proteins, such as human serum albumin, apoliproprotein, and fibrinogen, when adsorbed on nanoparticles is underway, which will help to address aspects of this problem [36,37]. The need to understand nanoparticles in a biological context is a pressing need that will determine the development of the exciting new fields of nanobiology, nanomedicine and nanotoxicology.
